# Wisconsin State Medical Society

**Published:** 1874-07-15

**Authors:** 


WISCONSIN STATE MEDICAL SOCIETY.—The regular annual meeting of this Society was held in Janesville, Wis., on the 16th, 17th, and 18th of June. Dr. Waterhouse presided, and the Profession of the State appeared to be well represented. A goodly number of papers were read, and topics of interest discussed, indicating a spirit of active inquiry creditable to the profession of the State. Dr. Henry Palmer, of Janesville, furnished the Society a very pleasant social entertainment at the Myer's House, which was highly enjoyed by about two hundred guests.

Papers or reports on the subject of medical education were read by Drs. D. Mason and J. B. Whiting, both advocating the exaction of a higher standard of education for medical students and practitioners. The Wisconsin State Society has taken the right position in regard to the preliminary education of medical students, and has a Board of Censors for the examination of such young men as propose to enter upon the study of medicine. It is not proper for any member of that Society to admit a student into his office without sufficient evidence of a fair general education. We hope the time will soon come when the profession in every State will adopt and strictly enforce a similar rule.

The officers elected for the ensuing year are as follows:

*President*, Dr. Reeve, of Appleton; *Vice Presidents*, Dr. E. W. Bartlett and Dr. L. D. Armstrong; *Secretary*, Dr. Theron Nichols, of Milwaukee; *Assistant Secretary*, Dr. Wm. Fox.

AN OVERLOOKED SOURCE OF BLOOD-SUPPLY, FOR TRANSFUSION IN POST-PARTUM HÆMORRHAGE.—Dr. William Highmore, writing to *The Lancet*, calls attention to the use, for purposes of transfusion, of the blood which has escaped from the open uterine vessels. By collecting and defibrinating this blood, and warming it to the requisite temperature, it may again be returned to the circulation of the patient, and enable the attendant to save a life in cases where an equally good source from which to obtain a supply of blood, does not exist.—*Med. Record.*

